# The Relationship of Serum 25-Hydroxyvitamin D and Insulin Resistance among Nondiabetic Canadians: A Longitudinal Analysis of Participants of a Preventive Health Program

**DOI:** 10.1371/journal.pone.0141081

**Published:** 2015-10-21

**Authors:** Truong-Minh Pham, John Paul Ekwaru, Sarah A. Loehr, Paul J. Veugelers

**Affiliations:** Population Health Intervention Research Unit, School of Public Health, University of Alberta, Edmonton, Canada; McMaster University, CANADA

## Abstract

Observational and intervention studies have revealed inconsistent findings with respect to the relationship between vitamin D and insulin resistance. No intervention studies have been conducted in community samples whereas this may be particularly relevant to the primary prevention of type 2 diabetes (T2D) and cardiovascular disease (CVD). In the present study we examined whether temporal improvements in vitamin D status, measured as serum 25-hydroxyvitamin D [25(OH)D], reduce the risk of insulin resistance among individuals without T2D. We accessed and analyzed data from 5730 nondiabetic participants with repeated measures of serum 25(OH)D who enrolled in a preventive health program. We used the homeostatic model assessment for insulin resistance (HOMA-IR) and applied logistic regression to quantify the independent contribution of baseline serum 25(OH)D and temporal increases in 25(OH)D on HOMA-IR. The median time between baseline and follow up was 1.1 year. On average serum 25(OH)D concentrations increased from 89 nanomoles per liter (nmol/L) at baseline to 122 nmol/L at follow up. Univariate analyses showed that relative to participants with baseline serum 25(OH)D less than 50 nmol/L, participants with baseline concentrations of “50-<75”, “75-<100”, “100-<125”, and ≥125 nmol/L were 0.76 (95% confidence intervals: 0.61–0.95), 0.54 (0.43–0.69), 0.48 (0.36–0.64) and 0.36 (0.27–0.49) times as likely to have insulin resistance at follow up, respectively. More importantly, relative to participants without temporal increases in 25(OH)D, those with increases in serum 25(OH)D of “<25”, “25-<50”, “50-<75”, “≥75” nmol/L were 0.92 (0.72–1.17), 0.86 (0.65–1.13), 0.66 (0.47–0.93), and 0.74 (0.55–0.99) times as likely to have insulin resistance at follow up, respectively. In the subgroup of participants without insulin resistance at baseline, this was 0.96 (0.72–1.27), 0.78 (0.56–1.10), 0.66 (0.44–0.99), and 0.67 (0.48–0.94), respectively. These observations suggest that improvements in vitamin D status reduce the risk for insulin resistance and herewith may contribute to the primary prevention of T2D and CVD.

## Introduction

The prevalence of type 2 diabetes (T2D) is increasing worldwide, and as such, it has become a global health issue [1]. The high prevalence of T2D can be attributed to several factors including aging of the population, poor lifestyles and excess body weight. According to the Public Health Agency of Canada [2], in 2011 approximately 7% of Canadians were living with T2D; however, this may represent an underestimation, as it does not consider those living with the undiagnosed condition [3]. Preventive strategies are essential to reduce the public health and economic burden of T2D [1]. The metabolic disorder whereby cells in the body do not respond to insulin properly is known as insulin resistance. It precedes the onset of T2D and thus is a functional target for the primary prevention of T2D. Insulin resistance is also implicated in the development of cardiovascular disease (CVD) [4].

Vitamin D is a steroid hormone that is synthesized in the skin by exposure to sunlight; it can also be obtained through diet and supplementation [5]. Vitamin D is changed into circulating 25-hydroxyvitamin D [25(OH)D], the nutritional biomarker for vitamin D status. The role of vitamin D in maintaining bone health has been well established [5], however, ubiquitous vitamin D receptors expressed in diverse cells throughout the body suggest that vitamin D also plays other important roles in health and disease. Associations between vitamin D status and chronic conditions have been extensively reported [6,7]; however, the relationship between vitamin D status and insulin resistance remains unclear.

Observational studies to date have revealed inconsistent findings with respect to the relationship between vitamin D status and insulin resistance [8–13]. Supplementation studies to date have focused on selected samples of subjects such as individuals with excess body weight [14], with existing insulin resistance and vitamin D deficiency (25(OH)D < 50 nmol/L) [15], with prediabetes [16] and with T2D [17,18], and also revealed inconsistencies in the effect of vitamin D on insulin resistance. No intervention studies, however, examined the potential benefits of vitamin D status for insulin resistance in community samples. Such studies are particularly relevant to establish the potential of vitamin D for the primary prevention of T2D and CVD. The present study aims to reveal whether prospective improvements in vitamin D status reduce the risk of insulin resistance among individuals free of T2D.

## Materials and Methods

### Study population

The Pure North S’Energy Foundation (PN) in Calgary, Alberta, Canada, is a not-for-profit organization that offers a preventive health program, of which details have been described elsewhere [19,20]. Briefly, the program was launched in October 2007 and employs health professionals who provide informed lifestyle counseling to volunteer participants. At enrollment, participants complete a lifestyle questionnaire, have a medical history and biometric measurements taken (height, weight, waist circumference, blood pressure), and have blood drawn for the assessment of serum 25(OH)D and other various biomarkers. The collected information is used to inform the health professionals as a basis for the participants’ lifestyle counseling. Vitamin D supplementation is encouraged given Canada’s Northern latitude, limited sunlight and cutaneous synthesis of vitamin D. Follow up visits for health assessments and lifestyle counseling are scheduled annually. The primary objective of the PN program is lifestyle counseling and disease prevention rather than scientific research; however, the PN does make their collected data available, in anonymized form, to the University of Alberta to allow for secondary data analysis. For that purpose, participants signed and granted written informed consent to allow their relevant information to be used for secondary data analysis. The ethical approval for the use of the PN data for research and scientific reporting was granted by the Human Research Ethics Board of the University of Alberta.

Data on 7,579 participants were obtained from the PN who were aged 18 years or older and had at least two measures for serum 25(OH)D concentrations, one at baseline and the other(s) at follow up visit(s) in order to examine the effect of temporal 25(OH)D changes on insulin resistance. Of these, we excluded 823 participants who reported being diagnosed with diabetes or were taking medications prescribed for diabetes. We also excluded 241 participants with fasting glucose concentrations ≥7 millimoles per liter (mmol/L), or elevated glycated hemoglobin (HbA1c ≥ 6.5%) because of suspicion of undiagnosed diabetes [3]. We further excluded three participants who had HOMA-IR values of 16 or higher [21]. In subgroup analyses we further excluded participants with insulin resistance at baseline. In our analyses we did not consider 205 participants who had fasted less than 8 hours prior to their blood work, 320 participants with missing serum insulin values, and 257 participants with either missing glucose values or suspicion of hypoglycemia (glucose concentrations < 3.5 mmol/L). This resulted in a total of 5730 participants with complete information.

### Measurements for serum 25(OH)D and other biomarkers

Serum 25(OH)D concentrations were measured by the DiaSorin LIAISON using the chemiluminescence immunoassay (CLIA) technology on an automated platform, and results were expressed as nanomoles per liter (nmol/L). The coefficient of variation of inter-assay for 25(OH)D measurements was 11%. Serum insulin concentrations were measured by the Abbott Architect on an automated immunoassay analyzer, and results were expressed as picomoles per liter (pmol/L) that were later divided by the constant of 6.945 to be converted into micro international units per milliliter (μIU/mL). The coefficient of variation of inter-assay for insulin measurements was 3%. Measurements for serum concentrations of glucose, triglycerides, total cholesterol, and high-density lipoprotein (HDL) cholesterol were performed by automated Roche Cobas 8000 Modular Analyzer Series. Results were expressed as mmol/L. The inter-assay CV was 1% for glucose, 2% for triglycerides, 1.5% for total cholesterol, and 2% for HDL cholesterol. Low-density lipoprotein (LDL) cholesterol concentrations were calculated from total cholesterol, and triglycerides according to the following formula: LDL cholesterol = Total cholesterol–HDL cholesterol–(Triglycerides /2.2)

### Homeostatic model assessment for insulin resistance (HOMA-IR)

We calculated HOMA-IR according to the equation described by Matthews et al. [22]: HOMA-IR = (FI x FG) / 22.5, where FI stands for fasting insulin (μIU/mL), and FG stands for fasting glucose (mmol/L). The denominator of 22.5 is a product of assumed normal fasting insulin (5 μIU/mL) and glucose (4.5 mmol/L) concentrations for typically healthy people [23]. Because there is no standard definition for insulin resistance, we defined it as HOMA-IR ≥ 2.73 according to the definition used in the National Health and Nutrition Examination Survey [24,25].

### Assessment for other variables

We obtained the following information on confounding variables from questionnaires completed by the participants: age, sex, season when blood samples were drawn, smoking habits (categorized as never smoker, past smoker, and current smoker), alcohol consumption (non-drinker, drinker), and physical activity. Body mass index (BMI) was computed as weight in kilograms divided by square of height in meters (kg/m^2^). Hypertension was defined as blood pressure equal to or greater than 140/90 mmHg or if a self-reported antihypertensive medication was currently being taken. Missing values for confounders were considered as missing categories in the regression analyses.

### Statistical analyses

Baseline serum 25(OH)D concentrations were considered as the following categories: “<50”, “50-<75”, “75-<100”, “100-<125”, and “≥125” nmol/L. We considered the lowest group with serum levels of “<50 nmol/L” as the referent group. Changes in serum 25(OH)D concentrations were calculated by subtracting the baseline concentration from the follow up concentrations, and were annualized by dividing it by the time (in years) between the baseline and follow up visit. Annualized changes were grouped into the following categories: “No improvement” (for values of 0 or less), “Increase of < 25”, “Increase of 25-<50”, “Increase of 50-<75”, and “Increase of ≥75” nmol/L. Baseline and follow up values for serum 25(OH)D, fasting insulin, glucose concentrations, HOMA-IR, and potential confounders were presented as descriptive statistics. As several participants had more than one follow up visit, we used the most recent follow up visit when presenting the descriptive statistics; however, we used all follow up information in all subsequent analyses. We first conducted cross-sectional analyses of baseline data by applying linear regression to quantify the change in HOMA-IR, fasting insulin and glucose concentrations for each 25 nmol/L increment in 25(OH)D. We then applied logistic regression to quantify the longitudinal relationship between baseline 25(OH)D and temporal changes in 25(OH)D concentrations and insulin resistance at follow up. These regression analyses were adjusted for the presence of insulin resistance at baseline, sex, age, season at baseline and at follow up when blood samples were drawn, baseline BMI, hypertension, LDL cholesterol, smoking status, drinking status, physical activity at baseline, and changes in physical activity from baseline to follow up. These logistic regression analyses were executed using generalized estimating equations methods to accommodate for the fact that some participants had more than one follow up visit.

All statistical analyses were performed using SAS 9.4 (SAS Institute Inc., Cary, USA). All statistical tests were two-sided and the level of statistical significance was at 0.05.

## Results

The baseline and follow up characteristics of the 5,730 nondiabetic participants, of whom 52% were women (n = 2975) and 48% men (n = 2755) are presented in Table 1. There were 3648 participants with one follow up visit, 1498 with two follow up visits, and 584 with three or more follow up visits. The median time between baseline and follow up was 1.1 year. The mean and median HOMA-IR did not change from baseline to follow up. The prevalence of insulin resistance (HOMA-IR ≥ 2.73) was 11% and 13%, respectively. The mean serum insulin concentrations decreased from 7.2 μIU/mL at baseline to 7.0 μIU/mL at follow up; while concentrations of serum glucose increased from 4.8 nmol/L to 5.0 mmol/L. On average, serum 25(OH)D concentrations increased from 89 nmol/L to 122 nmol/L from baseline to follow up. Other confounders did not change significantly from baseline to follow up with the exception of the number of participants that participated in a high level of physical activity, which increased from 30% to 33%, respectively.

**Table 1:** Baseline and follow up characteristics of 5730 study participants.

	Baseline	Follow up
**Serum 25(OH)D in nmol/L**	** **	** **
Mean (SD)	89 (42)	122 (46)
Median (IQR)	83 (31–109)	115 (89–148)
**Fasting insulin in** μ**IU/mL**		
Mean (SD)	7.2 (4.8)	7.0 (5.1)
Median (IQR)	6.0 (4.2–8.7)	5.6 (4.0–8.4)
**Fasting glucose in mmol/L**		
Mean (SD)	4.8 (0.5)	5.0 (0.5)
Median (IQR)	4.7 (4.4–5.1)	5.0 (4.6–5.3)
**HOMA-IR**		
Mean (SD)	1.6 (1.1)	1.6 (1.2)
Median (IQR)	1.2 (0.9–1.9)	1.2 (0.8–1.9)
**Insulin resistance, n (%)** ^a^	653 (11)	719 (13)
**Women, n (%)**	2975 (48)	2975 (48)
**Age, mean (SD)**	51 (15)	52 (15)
**BMI, n (%)**		
<18.5	58 (1)	54 (1)
18.5–<25.0	2019 (36)	2014 (35)
25.0–<30.0	2147 (38)	2128 (38)
> = 30.0	1443 (25)	1471 (26)
Missing	63	63
**Hypertension, n (%)** ^b^ ^,^ ^c^		
Normal	4353 (80)	3875 (78)
Elevated	1079 (20)	1081 (22)
Missing	298	774
**Serum LDL-cholesterol, n (%)** ^b^ ^,^ ^d^		
Normal	1911 (34)	1648 (30)
Elevated	3722 (66)	4001 (70)
Missing	97	81
**Season, n (%)**		
Winter	2349 (41)	2368 (41)
Spring	1552 (27)	1843 (32)
Summer	993 (17)	741 (13)
Fall	836 (15)	778 (14)
**Tobacco smoking status, n (%)** ^b^		
Never smoker	2357 (58)	1720 (58)
Past smoker	1243 (30)	863 (29)
Current smoker	483 (12)	375 (13)
Missing	1742	2772
**Alcohol drinking status, n (%)** ^b^		
Non-drinker	1422 (37)	1501 (38)
Drinker	2474 (63)	2416 (62)
Missing	1834	1813
**Physical activity, n (%)** ^b^		
Low	1577 (39)	1484 (36)
Moderate	1243 (31)	1315 (31)
High	1215 (30)	1366 (33)
Missing	1695	1565

25(OH)D, 25-hydroxyvitamin D; HOMA-IR, homeostatic model assessment for insulin resistance; LDL-cholesterol, low-density lipoprotein cholesterol; nmol/L, nanomoles per liter; μmol/L, micro moles per liter; μIU/mL, micro international units per liter; mmol/L, millimoles per liter; SD, standard deviation; IQR, interquartile range; BMI, body mass index.

^a^ Insulin resistance was defined as HOMA-IR ≥ 2.73

^b^ Percentage for these variables do not include missing observations

^c^ Hypertension was defined as blood pressure ≥140/90 mm Hg, or a self-report of taking antihypertensive medications

^d^ Elevated LDL-cholesterol was defined as LDL-cholesterol concentration ≥2.6 nmol/L.

Table 2 presents associations of fasting serum insulin, glucose concentrations, and HOMA-IR with 25(OH)D concentrations from cross-sectional analyses of baseline information. The β coefficients derived from linear regression analyses denote the average change in HOMA-IR, fasting insulin and glucose per each 25 nmol/L increment of 25(OH)D. For the entire sample, multivariable linear models showed that for every 25 nmol/L higher in 25(OH)D, values of both fasting insulin, HOMA-IR were significantly lower by 0.194, and 0.042, respectively; however, the change in fasting glucose was not significant. When these multivariable linear models were repeated separately by subgroup of baseline levels of 25(OH)D, we found that in individuals with 25(OH)D <50 nmol/L (14% of the participants), 25(OH)D concentrations were strongly associated with decreases in HOMA-IR, fasting insulin and glucose concentrations. Significant inverse associations between 25(OH)D concentrations and HOMA-IR and fasting insulin concentrations were also present in those with 25(OH)D ≥50 nmol/L (86% of the participants), however, these associations were less pronounced. No significant association was found for fasting glucose concentrations in this subgroup of 25(OH)D.

**Table 2:** Cross-sectional baseline associations of fasting insulin, glucose, and HOMA-IRwith 25(OH)D concentrations among 5730 study participants.

	Entire population (n = 5730)		25(OH)D <50 nmol/L (n = 801)		25(OH)D ≥50 nmol/L (n = 4929)	
	β coefficient (95% CI)	p	β coefficient (95% CI)	p	β coefficient (95% CI)	p
**Fasting insulin**						
Univariate	-0.464 (-0.531, -0.396)	<0.01	-1.161 (-2.234, -0.088)	<0.01	-0.387 (-0.459, -0.315)	<0.01
Multivariable	-0.194 (-0.256, -0.133)	<0.01	-1.351 (-2.315, -0.388)	<0.01	-0.144 (-0.207, -0.080)	<0.01
**Fasting glucose**						
Univariate	-0.015 (-0.023, 0.007)	<0.01	-0.178 (-0.288, -0.068)	<0.01	-0.010 (-0.019, 0.000)	0.05
Multivariable	0.003 (-0.005, 0.011)	0.47	-0.161 (-0.265, -0.055)	<0.01	0.006 (0.003, 0.015)	0.19
**HOMA-IR**						
Univariate	-0.107 (-0.123, -0.094)	<0.01	-0.311 (-0.562, -0.060	<0.01	-0.088 (-0.105, -0.071)	<0.01
Multivariable	-0.042 (-0.057, -0.028)	<0.01	-0.341 (-0.564, -0.119)	<0.01	-0.030 (-0.045, -0.015)	<0.01

25(OH)D, 25-hydroxyvitamin D; HOMA-IR, homeostatic model assessment for insulin resistance; nmol/L, nanomoles per liter; β coefficient (95% CI), coefficient derived from linear regression with 95% confidence interval; β coefficient quantifies the change in HOMA-IR, fasting insulin, and glucose per 25 nmol/L increment of 25(OH)D. Multivariable models were adjusted for sex, age, body mass index, hypertension, serum low-density lipoprotein cholesterol, seasons, smoking status, alcohol status, and physical activity.

Results of the longitudinal analyses of the risk of insulin resistance at follow up are presented in Table 3. Univariate analyses revealed that relative to participants with baseline serum 25(OH)D concentrations less than 50 nmol/L, those with baseline concentrations of “50-<75”, “75-<100”, “100-<125”, and “≥125” nmol/L were 0.76 (95% confidence intervals: 0.61–0.95), 0.54 (0.43–0.69), 0.48 (0.36–0.64), and 0.36 (0.27–0.49) times as likely to have insulin resistance at follow up, respectively. These estimates were less pronounced when adjusted for other variables. With respect to temporal increases in serum 25(OH)D concentrations, we found that relative to the individuals who did not have temporal increases in serum 25(OH)D concentrations, participants with an increase in serum 25(OH)D concentrations of “<25”, “25-<50”, “50-<75”and “≥75” nmol/L were 0.92 (0.72–1.17), 0.86 (0.65–1.13), 0.66 (0.47–0.93), and 0.74 (0.55–0.99) times as likely to have insulin resistance, respectively. Hypertension increased the risk of insulin resistance. Unlike smoking status, consumption of alcohol significantly decreased the risk for insulin resistance. Both physical activity at baseline and increases in physical activity from baseline to follow up reduced the risk for insulin resistance at follow up. As expected, overweight and obesity were strongly associated with risk of insulin resistance, of which the risks were 3.05 (2.22–4.19) and 9.16 (6.99–12.56), respectively.

**Table 3:** Risk for insulin resistance ^a^ among 5730 study participants with a total of 8752 follow up visits.

		Univariate		Multivariable	
	#visits	OR1 (95% CI)	p	OR2 (95% CI)	p
**Baseline 25(OHD), nmol/L**					
<50	1321	Reference		Reference	
50–<75	2420	0.76 (0.61–0.95)	0.02	0.86 (0.67–1.11)	0.25
75–<100	2212	0.54 (0.43–0.69)	<0.01	0.77 (0.58–1.04)	0.09
100–<125	1457	0.48 (0.36–0.64)	<0.01	0.72 (0.52–1.00)	0.05
> = 125	1342	0.36 (0.27–0.49)	<0.01	0.68 (0.47–0.99)	0.04
**Changes in 25(OH)D, nmol/L**	** **				
No improvement	1776	Reference		Reference	
Increase < 25	2687	1.09 (0.90–1.32)	0.38	0.92 (0.72–1.17)	0.50
Increase 25–< 50	1727	1.07 (0.86–1.33)	0.56	0.86 (0.65–1.13)	0.28
Increase 50–< 75	931	0.92 (0.71–1.19)	0.51	0.66 (0.47–0.93)	0.02
Increase > = 75	1631	1.08 (0.86–1.34)	0.51	0.74 (0.55–0.99)	0.04
**Baseline insulin resistance**	8752	18.8 (15.7–22.6)	<0.01	9.91 (8.11–12.12)	<0.01
**Men vs. women**	8752	1.49 (1.27–1.74)	<0.01	1.27 (1.05–1.54)	0.02
**Baseline age (per 10 years)**	8752	1.08 (1.03–1.13)	<0.01	1.03 (0.96–1.10)	0.40
**BMI at baseline** ^b^					
<18.5	87	0.54 (0.07–4.01)	0.54	0.41 (0.08–2.13)	0.29
18.5–<25.0	2974	Reference		Reference	
25.0–<30.0	3369	3.98 (2.94–5.39)	<0.01	3.05 (2.22–4.19)	<0.01
> = 30.0	2252	20.2 (15.2–27.0)	<0.01	9.16 (6.69–12.56)	<0.01
**Baseline hypertension** ^b^ ^,^ ^c^					
Normal	6587	Reference		Reference	
Elevated	1606	2.44 (2.05–2.91)	<0.01	1.45 (1.18–1.78)	<0.01
**Baseline LDL-cholesterol** ^b^ ^,^ ^d^					
Normal	2820	Reference		Reference	
Elevated	5774	1.04 (0.88–1.23)	0.68	0.84 (0.70–1.02)	0.08
**Season at baseline**					
Summer	1436	Reference		Reference	
Fall	1173	0.82 (0.62–1.08)	0.15	0.70 (0.50–0.97)	0.03
Winter	3799	0.80 (0.64–1.00)	0.05	0.54 (0.41–0.71)	<0.01
Spring	2344	0.91 (0.72–1.14)	0.42	0.67 (0.51–0.90)	0.01
**Season at follow up**	** **				
Summer	1378	Reference		Reference	
Fall	1329	0.72 (0.57–0.93)	0.01	0.70 (0.52–0.96)	0.03
Winter	3113	0.88 (0.73–1.07)	0.20	0.85 (0.67–1.08)	0.19
Spring	2932	1.12 (0.92–1.36)	0.26	1.20 (0.94–1.53)	0.14
**Baseline tobacco smoking status** ^b^					
Never smoker	3276	Reference		Reference	
Past smoker	1725	1.34 (1.10–1.64)	<0.01	1.07 (0.84–1.36)	0.58
Current smoker	601	1.26 (0.95–1.68)	0.11	1.00 (0.71–1.41)	0.99
**Baseline alcohol drinking status** ^b^					
Non-drinker	1902	Reference		Reference	
Drinker	3463	0.64 (0.53–0.76)	<0.01	0.71 (0.57–0.90)	<0.01
**Physical activity at baseline** ^b^	** **				
Low	2194	Reference		Reference	
Moderate	1706	0.65 (0.52–0.80)	<0.01	0.75 (0.58–0.96)	0.03
High	1663	0.33 (0.26–0.43)	<0.01	0.50 (0.38–0.67)	<0.01
**Physical activity change** ^b^					
No improvement	2038	Reference		Reference	
Moderate improvement	1336	0.95 (0.76–1.18)	0.64	0.68 (0.52–0.89)	<0.01
High improvement	766	0.74 (0.47–1.15)	0.18	0.66 (0.47–0.95)	0.02

25(OH)D, 25-hydroxyvitamin D; LDL-cholesterol, low-density lipoprotein cholesterol; OR (95% CI), odds ratio with 95% confidence interval; #visits, numbers of follow up visits; nmol/L, nanomoles per liter; BMI, body mass index.

^a^ Insulin resistance was defined as HOMA-IR ≥ 2.73

^b^ Missing data was considered in the analysis as a separate category

^c^ Hypertension was defined as blood pressure ≥140/90 mm Hg, or taking antihypertensive medications

^d^ Elevated LDL-cholesterol was defined as LDL-cholesterol concentrations ≥2.6 nmol/L.

OR1: Univariate analysis model.

OR2: Multivariable analysis model adjusted for variables presented in the table. Only changes in physical activity were taken into account for multivariate model, changes in BMI, tobacco smoking, and alcohol drinking were too minor to allow a meaningful analyses and therefore not included.

Table 4 presents results from analyses performed in Table 3 but with restriction to participants without insulin resistance at baseline. We observed that both higher baseline 25(OH)D concentrations and greater temporal increases in 25(OH)D concentrations during follow up were associated with a reduced risk of insulin resistance. Multivariable analysis showed that the risks of insulin resistance at follow up were 0.97, 0.84, 0.63, 0.53 for baseline 25(OH)D of “50–<75”, “75–<100”, “100–<125”, and “≥125” nmol/L, respectively, and 0.96, 0.78, 0.66, 0.67 for temporal increases in 25(OH)D of “<25”, “25–<50”, “50–<75”and “≥75” nmol/L, respectively, relative to the reference category (Table 4). Similarly, in an analysis which was further restricted to participants without vitamin D supplementation prior to baseline, risk of insulin resistance at follow up tended to be inversely associated with higher baseline 25(OH)D concentrations and greater increases in 25(OH)D concentrations during follow up, however, these associations were not statistically significant most likely because of the small number of visits.

**Table 4:** Risk ^a^ for insulin resistance ^b^ at follow up among participants without baseline insulin resistance.

	Without insulin resistance (n = 5095)			Without insulin resistance nor vitamin D supplementation (n = 1753)		
	#visits	OR (95% CI)	p	#visits	OR (95% CI)	p
**Baseline 25(OHD), nmol/L**						
<50	1075	Reference		441	Reference	
50–<75	2106	0.97 (0.71–1.32)	0.84	833	0.85 (0.52–1.38)	0.51
75–<100	2006	0.84 (0.59–1.20)	0.34	621	0.73 (0.42–1.26)	0.26
100–<125	1334	0.63 (0.42–0.97)	0.03	273	0.47 (0.22–1.02)	0.06
≥125	1270	0.53 (0.34–0.84)	0.01	178	0.95 (0.38–2.37)	0.92
**Changes in 25(OH)D compared with baseline, nmol/L**				** **		
No improvement	1612	Reference		409	Reference	
Increase of < 25	2398	0.96 (0.72–1.27)	0.76	599	0.92 (0.53–1.60)	0.77
Increase of 25–< 50	1542	0.78 (0.56–1.10)	0.16	476	0.70 (0.37–1.31)	0.27
Increase of 50–< 75	820	0.66 (0.44–0.99)	0.04	303	0.57 (0.29–1.15)	0.12
Increase of ≥ 75	1419	0.67 (0.48–0.94)	0.02	559	0.60 (0.33–1.10)	0.10

25(OH)D, 25-hydroxyvitamin D; OR (95% CI), odds ratio with 95% confidence interval; # visits, numbers of follow up visits; nmol/L, nanomoles per liter.

^a^ The analyses were adjusted for the variables included in the table, and additionally adjusted for gender, baseline body mass index, age, hypertension, serum low-density lipoprotein cholesterol, season at baseline, season at follow up, tobacco smoking status, alcohol drinking status, and physical activity at baseline, physical activity change during follow up

^b^ Insulin resistance was defined as HOMA-IR ≥ 2.73.

## Discussion

In the current longitudinal study of nondiabetic adults, we found that higher baseline and greater temporal increases in serum 25(OH)D concentrations were associated with a reduced risk for insulin resistance.

Inverse associations between serum 25(OH)D concentrations and insulin resistance have been reported in cross-sectional [8–10], prospective [13], and supplementation studies [14–16]. In a cross-sectional analysis of 1,204 adult participants from a Jerusalem birth cohort [8], a modest inverse association between serum 25(OH)D concentrations and the logarithm of HOMA-IR in men was reported, but this association was not significant in women. A cross-sectional analysis of 712 nondiabetic participants from the Prospective Metabolism and Islet cell Evaluation study in Canada [10] revealed a significant association between the logarithm of HOMA-IR and serum 25(OH)D concentrations. The National Health and Nutrition Examination Survey showed that low concentrations of 25(OH)D were associated with insulin resistance in a cross-sectional analysis among 3,206 nondiabetic participants [9]. Where the aforementioned studies and the cross sectional analyses of the present study did reveal an association, two other cross-sectional studies had failed to demonstrate independent associations [11,12]. A 10 year prospective cohort study of 524 nondiabetic subjects in United Kingdom [13] reported an inverse association between baseline 25(OH)D concentrations and insulin resistance, consistent with the findings of the present study. Supplementation studies among individuals with excess body weight or prediabetes [14,16] failed to demonstrate an effect of vitamin D supplementation on insulin resistance, whereas a randomized controlled trial among vitamin D deficient women (serum 25(OH)D concentrations <50 nmol/L) with insulin resistance did report a reduction in the risk for of insulin resistance along with improvements in serum 25(OH)D concentrations [15]. The present study also showed decreases in the risk for insulin resistance with increases in serum 25(OH)D concentrations. With respect to studies among T2D patients, Wallace et al [17] reported that none of the five supplementation studies had shown an effect of vitamin D on insulin resistance with the exception of a subgroup of T2D patients who had experienced more pronounced increases in serum 25(OH)D concentrations. The effect of supplementation on vitamin D status is not straightforward. Improving serum 25(OH)D concentrations have been shown to require 2 to 3 times higher vitamin D supplementation doses for obese subjects than for normal weight subjects [19]. This complicates the interpretation of supplementation studies and points at the benefits of studying serum 25(OH)D concentrations, as we did in the present study.

Studies examining the effect of serum 25(OH)D on the development to T2D have also produced inconsistent results. Although prospective studies, including the Framingham Offspring Study [26] and the Nurses’ Health Study [27] reported that higher 25(OH)D concentrations were associated with a decreased risk of T2D, randomized controlled trials of vitamin D supplementation failed to reveal a significant effect on the progression of new diabetes [18]. The latter study also highlighted how few trials have been conducted among healthy subjects and in population-based samples. Clearly, such studies are essential to justify investments in primary prevention.

In the present study, baseline analysis provided a cross-sectional relationship between serum 25(OH)D concentrations and HOMA-IR, fasting serum insulin, and glucose concentrations (Table 2). This analysis was comparable with previous cross-sectional studies [8–10] in terms of analytical approach. Multivariable analysis showed that each 25nmol/L increment in 25(OH)D concentrations was inversely associated with HOMA-IR and fasting insulin concentrations, but not with fasting glucose concentrations. This suggests that vitamin D status presumably affects insulin resistance through reduced insulin. The sub-analysis amongst participants with vitamin D deficiency (<50nmol/L) revealed a more pronounced association between 25(OH)D and HOMA-IR. This adds to the importance of prevention for vitamin D deficiency.

The development of insulin resistance is multifactorial. Our results contributed additional evidence towards relationships between insulin resistance and previously suggested lifestyle factors such as physical activity and weight status. In the present study we revealed inverse associations between both vitamin D concentrations at baseline and prospective improvements in vitamin D status at follow up with insulin resistance, independent of physical activity and weight status. Indeed, overweight and obesity have been recognized as predictors for insulin resistance through dysfunction of adipocytes [28,29], whereas physical activity has been known as an important factor for the improvement of insulin sensitivity [30,31]. Also age is an important factor in the development of insulin resistance. The aging of the study population in combination with the fact that this was a volunteer program and not all participants increased their serum 25(OH)D concentrations may have contributed to the fact that mean serum concentration of HOMA-IR did not change and the prevalence of insulin resistance increased during follow-up. Moderate alcohol consumption has been previously reported to improve insulin sensitivity [32], and consequently may further contribute to the lower risk of cardiovascular disease [33]. Therefore, actions from the point-of-view of public health implementation would benefit from the findings of the present study. Specifically, vitamin D supplementation, encouragement of physical activity, and weight management may together contribute to improve insulin resistance and lessening the potential for the development of T2D.

The underlying mechanisms by which vitamin D decreases the risk of insulin resistance have yet to be elucidated. Several pathways have been suggested to be involved. Vitamin D deficiency has been reported to be associated with an increase in parathyroid hormone, which in turn is associated with decreased insulin sensitivity [34]. In addition, vitamin D regulates calcium homeostasis and has a role in maintaining adequate intracellular levels of calcium for intracellular process in insulin-responsive tissues such as at muscle cells [35]. Furthermore, vitamin D receptors are found in pancreatic β-cells, and vitamin D is involved in some of the biological activities of insulin including normal insulin secretion, stimulation of the expression of insulin receptors, and increasing insulin responsiveness for glucose transport [36,37].

The longitudinal design, the large sample size, and the wide range of serum 25(OH)D concentrations are strengths of the present study. Limitations of the current study should also be discussed. First, the glucose clamp technique [23,38], the “gold standard method” for quantifying insulin resistance, was not performed. This technique is time and cost intensive, and usually performed in research settings rather than in clinical practice like the PN program. Instead, we used HOMA-IR as a surrogate index of insulin resistance. The two methods, however, are highly correlated [39]. Second, although we adjusted for important confounders, the possibility of potential bias due to unmeasured confounders should not be ruled out. The lack of data on parathyroid hormone must especially be noted since this hormone is reported as a mediator for glucose metabolism, as well as interferes with pancreatic cell function [40]. Third, the present study is an analysis of secondary data of a volunteer program without random allocation and blinding. For the aforementioned reasons we recommend the relation between vitamin D status and insulin resistance in healthy subjects be further studied in randomized controlled trials.

In summary, the results from the present study suggest that prospective improvements of vitamin D status decrease the risk of insulin resistance in participants without insulin resistance or T2D. In Canada where 35% of adults are vitamin D deficient [41], improving the vitamin D status in the population, along with promotion of physical activity and healthy body weights, may reduce the prevalence of insulin resistance and contribute to the primary prevention T2D and CVD.
